# DNA–dependent protein kinase in telomere maintenance and protection

**DOI:** 10.1186/s11658-020-0199-0

**Published:** 2020-01-17

**Authors:** Jiangdong Sui, Shichuan Zhang, Benjamin P. C. Chen

**Affiliations:** 1 Radiation Oncology Center, Chongqing University Cancer Hospital, Chongqing, 400030 China; 2 Department of Radiation Oncology, Sichuan Cancer Hospital, Chengdu, China; 3 Department of Radiation Oncology, University of Texas Southwestern Medical Center, 2201 Inwood Rd., Dallas, TX 75390–9187 USA

**Keywords:** DNA–PK, Shelterin, Telomerase, Telomere, hnRNP–A1

## Abstract

This review focuses on DNA–dependent protein kinase (DNA–PK), which is the key regulator of canonical non–homologous end–joining (NHEJ), the predominant mechanism of DNA double–strand break (DSB) repair in mammals. DNA–PK consists of the DNA–binding Ku70/80 heterodimer and the catalytic subunit DNA–PKcs. They assemble at DNA ends, forming the active DNA–PK complex, which initiates NHEJ–mediated DSB repair. Paradoxically, both Ku and DNA–PKcs are associated with telomeres, and they play crucial roles in protecting the telomere against fusions. Herein, we discuss possible mechanisms and contributions of Ku and DNA–PKcs in telomere regulation.

## Introduction

DNA–dependent protein kinase (DNA–PK) consists of the DNA–binding Ku70/80 heterodimer and the catalytic subunit DNA–PKcs. It is the key regulator of the canonical non–homologous end–joining (HNEJ) mechanism for DNA double–strand break (DSB) repair. The Ku heterodimer, which is highly abundant in mammals, has an extremely high affinity for broken DNA ends, with its ring–shaped structure easily sliding into place. The loading of Ku at the DNA ends quickly recruits DNA–PKcs to form the active DNA–PK complex, which is essential for NHEJ–mediated end–joining activity (see references in [1]). The catalytic DNA–PKcs subunit is a member of the phosphatidylinositol–3 kinase–like kinase (PIKK) family, which includes ataxia–telangiectasia mutated (ATM) and ATM– and Rad3–related (ATR). Together, they are key upstream kinases in sensing DNA damage and promoting DNA damage repair to preserve genome integrity [2, 3].

Telomere maintenance is crucial to protect the integrity of linear chromosomes in eukaryotes. Mammalian telomeres, which have highly repetitive TTAGGG sequences with a single–stranded, G–rich extension (“overhang”) at the 3′ ends, are protected by the shelterin protein complexes [4, 5]. These contain six subunits, including the double–stranded telomeric DNA–binding factor TRF1/2 and the single–stranded telomeric DNA–binding factor POT1. They enable the formation of the t–loop structure where the single–stranded telomeric overhang hides inside the duplex part of the telomere to prevent the recognition of telomere ends by the DNA repair machinery [6]. The critical issues of telomere maintenance mostly occur during the transition between DNA replication and reestablishment of the t–loop telomeric capping structure to guard the G–rich 3′ overhangs. Furthermore, DNA replication cannot be completed at the very end of the telomere since the lagging strand replication requires upfront synthesis of Okazaki fragments. Telomere shortening, which is inevitable through each cell division, results in replicative senescence [7, 8]. Telomerase and the alternative lengthening of telomeres (ALT) mechanism evolved to extend telomere length and counterbalance telomere shortening during each cell cycle. Such telomere expansion strategies are crucial for continuous expansion of stem cell populations, although they also contribute to cancer development [7, 9]. For example, the ALT mechanism, which is dependent on homologous recombination (HR), is involved in roughly 10–15% of human cancers [10].

The NHEJ mechanism downstream from the DNA–PK complex is responsible for fusion of unprotected telomeres. Interestingly, Ku and DNA–PKcs are required for telomere protection at multiple steps. In this review, we focus on their participation and mechanism in this crucial process for chromosome integrity.

## Association of DNA–PK with the shelterin complex

The repetitive TTAGGG sequences of mammalian telomeres are primarily protected by the specialized six–subunit shelterin complex, which comprises TRF1, TRF2, POT1, TPP1, TIN2 and RAP1 [4, 5]. This complex guarantees the stability of the t–loop structure to shelter the telomeres against a series of harmful situations [5]. TRF1 and TRF2 are connected by their association with TIN2, and they abundantly bind to the duplex part of telomeres with distinct roles in telomeric protection. TRF1 facilitates efficient telomeric replication and prevents replication fork stalling by recruiting and/or activating a class of helicases [11, 12]. TRF2 promotes the maintenance of the telomeric overhang by recruiting the Snm1b/Apollo nuclease to the newly replicated blunt–ended leading–strand telomeres and prevents telomeric overhang degradation by nucleases [13–15]. It also protects the telomere against fusions in part by counteracting ATM kinase activation and thus suppressing DDR signaling at telomeres [16–18]. Similarly, POT1, which binds specifically to the single–stranded telomeric DNA, represses ATR kinase activation at telomeres [19].

Ku and DNA–PKcs have been found to independently associate with different shelterin complex components. The DNA–binding ku70/80 heterodimer is able to interact physically with TRF1, TRF2 and RAP1 [20–22]. Ku is known to have a very high affinity to all DNA termini regardless of the sequences, although it appears that Ku does not bind to duplex telomeric DNA directly but tethers with TRF1 to bind indirectly [23]. Such indirect binding of Ku could contribute to the inhibition of NHEJ activity at telomeres [24, 25]. The catalytic DNA–PKcs is able to interact with TRF2 and RAP1 at telomeres, and their association prevents end–joining [17]. Additionally, the DNA–PKcs–interacting protein KIP/CIB is required to mediate DNA–PKcs recruitment to telomeres and bridge the association between DNA–PKcs and TRF2 for telomere protection [16]. These results suggest that the DNA–PK complex is recruited to the internal region of the telomeres rather than the very end, and that it participates in telomeric maintenance through TRF1 and TRF2.

## DNA–PK on modulation of telomerase activity

Telomerase is a ribonucleoprotein complex that contains a catalytic telomerase reverse transcriptase (TERT) subunit and an integral telomerase RNA component (Terc, also referred to as TR, telomerase RNA) subunit for telomere maintenance and elongation [26–29]. TR is required to serve as a template for the synthesis and extension of the G–rich 3′ telomeric overhang by TERT [26]. The regulation of telomerase activity at telomeres is complex and involves several accessory factors associated with TERT, including Ku [30]. It was reported that telomerase was co–immunoprecipitated with antibodies against Ku in human cells, and that Ku physically interacts with in vitro translated human TERT in the absence of human TR (hTR) and telomeric DNA [31]. Studies from yeast Ku indicate that it interacts with telomerase–associated TLC1 RNA (yeast TR) and Cdc13, which recognizes single–strain telomeric DNA [32–35]. In fact, yeast Ku is capable of binding to the stem–loop structure of TLC1 RNA [33, 34], and facilitates nuclear retention of TLC1 critical for telomere homeostasis [35]. This is consistent with the studies that human Ku could bind directly to hTR and elicit DNA–PK kinase activity [36, 37]. Mutation analysis revealed that the same DNA–binding surface of yeast Ku80 is required for interactions with TLC1 and that it facilitates telomerase recruitment to telomeres [38]. Furthermore, the expression of a Cdc13–Ku70 fusion protein leads to telomeric extension [32]. Additional protein–protein associations among yeast Ku70/80, telomeric transcriptional silencing protein Sir4 and Rap1 likely also contribute to telomerase recruitment [39–41]. These results show that Ku plays a crucial role in promoting or stabilizing telomerase to the telomeric DNA in yeast for telomere maintenance.

The involvement of DNA–PKcs in telomerase regulation is less clear. It is able to form a protein complex with TERT through its interacting protein KIP, and overexpression of KIP improves telomerase activity in human cells [42]. Conversely, it was reported that hTR interacts with the Ku heterodimer and stimulates DNA–PK kinase activity on heterogeneous nuclear ribonucleoprotein A1 (hnRNP–A1), which binds to single–strand telomeric DNA and plays a critical role in telomere biogenesis [36, 37, 43]. Mouse genetics analyses reveal that in telomerase deficient background (Terc^−/−^), disruption of Ku or DNA–PKcs genes result in progressively shorter telomeres [44, 45], suggesting that the DNA–PK complex coordinates with telomerase to preserve normal telomeres.

## Implication of DNA–PK for telomere length regulation

The function of telomeric capping for the DNA–PK complex is superficially paradoxical in light of its role in promoting the NHEJ pathway. This probably reflects its distinct roles at telomeric versus broken ends. Multiple studies from different groups suggested that all three subunits of the DNA–PK complex contribute to telomeric capping protection, since deficiency in either subunit results in increased incidents of telomere fusion in mouse and human cells (see further discussion below). It is less clear whether the DNA–PK complex contributes to the maintenance of telomere length. While loss of Ku results in telomere shortening in most eukaryotes, telomeric expansion was found in *Drosophila* and *Arabidopsis* in the absence of Ku [46]. It is possible that Ku is required to restrict telomere lengthening through telomerase or HR–mediated ALT mechanisms, particularly in those eukaryotic species where HR is the predominant type of DSB repair.

Knockout of the mouse Ku86 gene causes the accumulation of telomere fusions but there are conflicting reports regarding telomere length regulation, with either shortening or lengthening being shown [23, 44, 47, 48]. In the absence of telomerase activity (in a Terc^−/−^ background), knockout of Ku86 results in progressively shorter telomeres in later generations of Terc^−/−^/Ku86^−/−^ mice [44].

Loss of DNA–PKcs in mice also lead to mixed reports on telomere length regulation [45, 49]. In the absence of telomerase activity, DNA–PKcs deficiency accelerates telomere shortening even in the first generation of Terc^−/−^/DNA–PKcs^−/−^ double knockout mice. This is accompanied by decreased proliferation of germ cells, contrasting to the development of these phenotypical defects in later generations in Terc^−/−^ mice [45, 50]. These results demonstrate an accelerated rate of telomeric shortening in the absence of telomerase and the DNA–PK complex. They suggest that the DNA–PK complex in association with telomerase does play a role in telomere length maintenance.

## Implication of DNA–PK on telomeric capping

### The role of the Ku heterodimer on telomeric capping

It is apparent that Ku is involved in telomere length modulation in all eukaryotic species [46]. The evidence for its role in telomeric capping and prevention of fusion came from studies in mouse and human cells. It is speculative that its participation in telomeric capping protection is restricted in higher organisms or only found in vertebrates. Ku is clearly crucial in protecting telomeres from end–to–end fusions in mouse cells since Ku knockout increases the frequency in telomeric fusions [47, 51]. However, Ku also appears to promote telomere fusions when telomeres are critically shortened in telomerase–deficient mouse cells [44]. These results indicate that critical telomere length and telomeric interacting proteins are necessary to modulate Ku activity in telomere protection or end–to–end fusions via the NHEJ mechanism. Mutation analyses reveal that the helix 5 (α5) of yeast Ku70 has a selective impact on NHEJ, whereas mutations in the α5 of yeast Ku80 have a selective impact on telomeric maintenance. A spatially organized ‘two–face’ model of the Ku heterodimer was proposed with an outward Ku70 NHEJ–specific α–helix surface dealing with DSB repair and an inward Ku80 telomeric silencing α–helix dealing with telomeric regulation [24].

Notably, TRF2 was reported to interact with Ku70 in a way that involved α5, suggesting a mechanism by which TRF2 can impedes the NHEJ function of Ku on synapsing telomere ends [25]. This is consistent with the report that TRF2 can remodel telomeric DNA into t–loop configurations to block the end–loading of the Ku heterodimer, in turn preventing telomeres from engaging in Ku–dependent NHEJ [52, 53]. The distinctive features of yeast Ku70 and Ku80 are conserved in mouse and human Ku proteins. The same mechanism is likely to apply to mammalian Ku proteins in telomeric capping and protection, although further validation is needed. Additionally, mouse Ku cooperates with TRF2 and POT1 to prevent sister telomere exchanges mediated by HR–dependent recombination between sister telomeres [53, 54].

Knockout of mouse Ku70 or Ku86 is associated with retarded growth, dwarfism and premature aging characteristics, but it does not cause developmental lethality [55–58]. On the contrary, partial deletion of Ku by siRNA or inactivation of a single allele of Ku in human cells leads to increased apoptosis and severe loss of telomere integrity, including telomere fusions and length shortening [59–61]. Furthermore, complete elimination of both copies of the Ku86 gene results in somatic lethality and massive telomere loss in the form of open circular telomeric DNA [62]. These studies demonstrate that the Ku heterodimer is essential for telomere maintenance and cell viability in humans.

### Involvement of DNA–PKcs kinase activity and its Thr2609 cluster on telomeric capping

Although Ku proteins are evolutionarily conserved and required for telomere protection in all eukaryotic species, DNA–PKcs homologs are primarily found in vertebrates. Information on the role of DNA–PKcs in telomere protection was mostly generated in mouse or human cells. DNA–PKcs deficiency occurs naturally in mice, dogs and horses and results in the severe combined immunodeficiency (SCID) phenotype [63–67]. Investigation of SCID mouse cells revealed an increase in spontaneous chromosome aberrations including both chromosome– and chromatid–type telomere fusions, suggesting that DNA–PKcs plays an important role in telomeric capping [51]. Similar conclusions were subsequently validated in genetically engineered DNA–PKcs null or mutant mouse models [68–72]. Furthermore, specific leading–to–leading chromatid–type telomere fusions were reported in DNA–PKcs^−/−^ mouse cells [45, 70] and in DNA–PKcs^3A/3A^ mouse cells defective in DNA–PKcs Thr2609 cluster phosphorylation [71] (see below for further discussion).

**Fig. 1:**
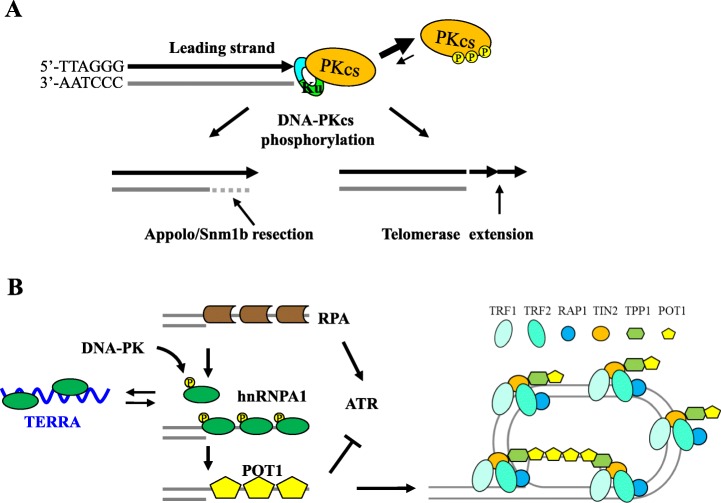
DNA–PK facilitates G–overhang production and telomeric capping. **a** DNA–PKcs phosphorylation at the Thr2609 cluster promotes dissociation of the DNA–PK complex at newly replicated blunt–ended leading telomeres. This enables G–overhang production through i) telomerase–mediated telomere extension, or ii) Snm1b/Apollo exonuclease–mediated end–resection. **b** DNA–PK–dependent hnRNP–A1 phosphorylation improves hnRNP–A1’s ability to displace RPA and favor POT1 loading at single–stranded telomeric DNA. This supports T–loop formation. TERRA negatively regulates T–loop formation by trapping hnRNP–A1 away from telomere overhangs. The RPA–to–POT1 displacement also prevents ATR signaling activation at single–stranded telomeric DNA

Thus, DNA–PKcs could play an important role in processing the blunt–ended leading–strand telomeres after DNA synthesis to produce the single–stranded G–overhangs (Fig. 1a), whereas the overhangs occur naturally at the lagging strand and shield themselves following replication [73, 74]. Such leading–to–leading telomere fusions were not reported in Ku70 or Ku80 knockout mouse cells. It is possible that Ku and DNA–PKcs contribute to the telomeric capping and maturation process in distinct ways. For example, DNA–PKcs is required to resolve the stalled replication fork in telomeres and participates in replication stress signaling independently of Ku [75, 76].

Significant loss of telomere protection, but not telomere shortening, was found in post–replicated leading telomeres in DNA–PKcs^3A/3A^ mutant mouse cells lacking a functional DNA–PKcs Thr2609 phosphorylation cluster [71, 77]. The Thr2609 cluster is crucial for DNA–PKcs activity in DSB repair and radiation resistance [78–80]. Although it was initially identified as an autophosphorylation event, subsequent analyses revealed that the Thr2609 cluster is respectively targeted by ATM and ATR kinases in response to DSBs and replication stress [79, 81]. Consequently, DNA–PKcs phosphorylation at the Thr2609 cluster triggers a series of conformational changes and modulates the dynamic association and dissociation of the DNA–PK complex at DNA termini [82, 83].

The importance of the DNA–PKcs Thr2609 cluster was further demonstrated using DNA–PKcs^3A^ mutant mice harboring three alanine substitutions to eliminate DNA–PKcs phosphorylation at the Thr2609 cluster. Homologous DNA–PKcs^3A/3A^ mice all die prematurely after birth due to the loss of hematopoietic stem cells (HSCs) and congenital bone marrow failure, which are not found in DNA–PKcs null or SCID mice [77]. Furthermore, DNA–PKcs^3A/3A^ cells displayed significant telomere fusions without apparent telomere shortening. Approximately 46% of DNA–PKcs^3A/3A^ metaphase spreads display telomere abnormalities compared to 20% in DNA–PKcs^−/−^ and 1% in DNA–PKcs^+/+^ metaphase spreads [71]. Similarly, high incidents of telomere fusions were also found in kinase dead DNA–PKcs^KD/KD^ mouse cells [72], or upon treatment with DNA–PKcs kinase inhibitors [84, 85].

These results suggest that DNA–PK kinase activity and Thr2609 cluster phosphorylation are crucial for telomere protection. Whether DNA–PK kinase inhibition impairs Thr2609 cluster phosphorylation and telomere deprotection is not clear since ATM and ATR also contribute to DNA–PKcs Thr2609 cluster regulation in vivo. Nonetheless, DNA–PK kinase activity is elicited during G2/M phases and necessary for Thr2609 cluster phosphorylation [86]. DNA–PK kinase activation is probably required to modulate additional telomere regulators such as hnRNP–A1 (see below for further discussion) and Werner (Wrn) syndrome protein to assist in telomere capping development. It was reported that DNA–PKcs stimulates Wrn helicase activity (but not its exonuclease activity) to unwind and release the D–loop substrate, and that overexpression of Wrn reversed telomeric G–overhang shortening in DNA–PKcs knockdown cells [87].

Significant and spontaneous γH2AX signals were observed specifically in mitotic DNA–PKcs^3A/3A^ cells from cell culture and tissue analyses. Furthermore, these mitotic γH2AX signals predominantly occur at leading–strand telomeres [71]. The newly synthetized leading–strand telomeres are nearly blunt–ended or carry a few nucleotides in overhang, and require a G2/M phase processing event for final maturation of the G–overhang [74, 88]. The leading G–overhang can be produced through telomerase–dependent telomere elongation [73, 74]. Alternatively, it can be generated through an end–resection by Snm1b/Apollo, a TRF2–interacting exonuclease involved in production of leading overhangs after replication and protection of leading telomeres from engagement with NHEJ–mediated repair [14, 15].

By contrast, lagging–strand telomeres form the G–rich overhangs automatically due to the removal of the RNA primer in the terminal Okazaki fragment and failure to position the fragment at the chromosome terminus. Leading strand–specific γH2AX signals caused by DNA–PKcs^3A^ mutant protein suggest that DNA–PKcs and the Ku heterodimer are present leading telomeric ends immediately after replication. Subsequent DNA–PKcs phosphorylation and conformational change triggers DNA–PKcs dissociation from the very end of leading telomeres [82, 83]. This dynamics allows an orderly processing of G–overhang at the leading daughter telomeres, otherwise the prolonged occupancy by the mutant DNA–PK^3A^ protein complex interferes with overhang production at leading telomeres, elicits DDR signaling, and results in loss of HSCs and presaging phenotypes [71, 77]. This hypothesis predicts that, upon removal of the Ku heterodimer, DNA–PKcs^3A^ mutant protein alone cannot disrupt overhang synthesis at the leading–strand telomeres. Indeed, the Ku86^−/−^/DNA–PKcs^3A/3A^ double mutant mice survive much longer than DNA–PKcs^3A/3A^ mice (BC unpublished result). It is also possible that DNA–PKcs phosphorylation influences its interaction with TRF2 or the ability of TRF2 to recruit the Snm1b/Apollo exonuclease to leading telomeres [14–17].

The short lifespan and HSC loss characters of DNA–PKcs^3A/3A^ mice can be rescued by bone marrow transplantation (BMT) although the BMT–rescued DNA–PKcs^3A/3A^ mice are prone to cancer at multiple sites including high incidence of skin squamous cell carcinoma (SCC) and lymphoma [71, 77]. This indicates that a functional DNA–PKcs T2609 cluster is required for proper maintenance of telomeres to prevent genomic instability and cancinogenesis.

In support of this, a DNA–PKcs Thr2609Pro mutation was previously identified from a breast cancer biopsy [89]. Expresion of Thr2609Pro mutant DNA–PKcs protein resulted in leading–strand telomeric deprotection as shown in DNA–PKcs^3A/3A^ mouse cells [71]. The DNA–PKcs^3A^ mouse model resembles dyskeratosis congenita (DC), a rare bone marrow failure syndrome that is characterized by defects in telomere maintenance [90, 91]. DC patients are known to be at high risk of developing head and neck SCC and hematologic malignancies [92]. Considering the phenotypical similarity, it is reasonable to speculate that mutations in the DNA–PKcs *PRKDC* gene could be found in DC patients.

### DNA–PK–dependent hnRNP–A1 phosphorylation facilitates telomeric capping

The main challenges in telomere maintenance occur during the transition between DNA replication and reestablishment of telomeric capping protection. The newly synthesized G–overhangs are protected by the replication protein A (RPA) complex, which is the predominant single–stranded DNA–binding (ssDNA–binding) protein and is essential for both DNA replication and damage repair [93]. An extended ssDNA–RPA filament at stalled replication forks will trigger the ATR–Chk1 S–phase checkpoint pathway and promote DNA repair [94, 95]. Thus, it is critical that POT1 rapidly displaces RPA at newly synthetized telomeric overhangs to prevent unnecessary DDR. POT1 is the main single–stranded telomeric DNA–binding factor of the shelterin complex, but it cannot out–compete RPA on its own: it requires additional support from hnRNP–A1 [96]. HnRNP–A1 is versatile factor involved in multiple processes during RNA biogenesis and a critical regulator of telomere homeostasis [97, 98]. It is capable of binding to single–stranded telomeric DNA and the RNA component of telomerase. It also promotes telomerase activation and telomere length extension [99, 100].

Since hnRNP–A1 is the direct substrate of DNA–PK kinase, its role in telomere protection has been linked to DNA–PK [37, 101]. HnRNP–A1 phosphorylation by DNA–PK in vivo coincides with telomeric overhang synthesis during G2/M phases. Consequently, hnRNP–A1 phosphorylation promotes its ability to bind to single–stranded telomeric DNA and facilitates the RPA–to–POT1 switch [43]. Conversely, cells lacking hnRNP–A1 or expressing the phospho–dead mutant hnRNP–A1 display an elevated γH2AX signal at telomeres and higher incidents of telomere aberrations, including sister telomere fusions [43]. How hnRNP–A1 phosphorylation improves its ability to bind to telomeric DNA is not clear. It is notable that hnRNP–A1 Ser95, one of the two key phosphorylation residues, is located between the RNA– and DNA–binding RRM1 and RRM2 motifs, suggesting that phosphorylation induces a conformational change to improve their access to RNA and DNA. Alternatively, DNA–PKcs–dependent hnRNP–A1 phosphorylation could modulate the intermolecular dimerization of hnRNP–A1 and affect its RNA– and DNA–binding ability [102]. These results demonstrate that DNA–PK kinase activity promotes the RPA–to–POT1 switch through hnRNP–A1 phosphorylation to facilitate telomeric capping protection (Fig. 1b).

The ability of hnRNP–A1 to bind to single–stranded telomeric DNA is modulated by telomeric repeat–containing RNA (TERRA), the non–coding RNA species produced from the sub–telomeric region by RNA Pol–II–mediated transcription [103]. The direct interaction between hnRNP–A1 and TERRA could trap hnRNP–A1 away from telomeric overhangs to promote the RPA–to–POT1 switch. Nonetheless, the abundance of TERRA peaks during G1 and decreases gradually from S phase to mitosis, thus releasing the TERRA–bound hnRNP–A1 to compete with RPA and promote POT1 loading to telomeric overhangs [96, 104]. These findings suggest that the balance between hnRNP–A1 and TERRA is crucial for telomere homeostasis and telomerase activity, since excessive TERRA molecules prevent telomere extension by telomerase and the RPA–to–POT1 switch, whereas excessive hnRNP–A1 proteins could overload telomeric overhangs and prevent their access to telomerase or POT1 [105]. It is interesting to note that DNA–PKcs has been identified among TERRA RNA–binding proteins [106]. Considering its weak DNA affinity [107], it is unlikely that DNA–PKcs binds to TERRA directly but is rather tethered to it through other TERRA–binding proteins. In addition, its ability to phosphorylate hnRNP–A1 and to regulate RNA Pol–II transcription could potentially influence TERRA production and regulation in telomere maintenance [43, 108].

## DNA–PK coordinates with topoisomerase–II to resolve stalled replication fork at telomeres

**Fig. 2:**
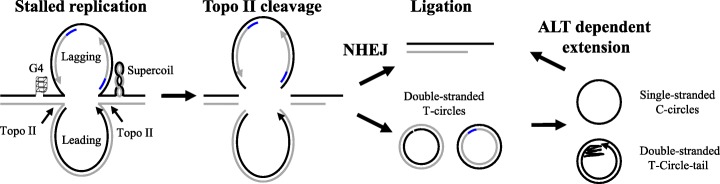
A “looping–out” mechanism to resolve a stalled replication fork at telomeres via the topoisomerase II (Topo–II) and NHEJ mechanism. Unresolved G–quadruplex (G4) structures hinder completion of DNA replication at telomeric regions. Topo–II cleavages on both sides release the stalled replication fork and generate both leading and lagging daughter DNA. Repair and ligation via the NHEJ, HR or other repair mechanisms support replication, which resumes at newly ligated telomeres, causing the production of T–circles from the released leading and lagging DNA. The T–circles progress into T–circle–tail or single–stranded C–circles, which could participate in telomere extension through the ALT mechanism. This figure is modified from Zeng et al., EMBO Rep 18: 1412–1428

The G–rich and repetitive nature of telomere is prone to G–quadruplex secondary structure formation, which blocks the progression of DNA replication fork and induces replication stress [109]. For example, treatment with TMPyP4, a stabilizer of G–quadruplex, induces severe replication fork stalling at the telomere and results in significant production of fragile telomeres [110]. Resolution of replication stress at telomeres leads to the formation of circular telomeric DNA with a single–stranded tail or the “t–circle–tail” structure, mediated by topoisomerase II (Topo–II) and DNA–PK–mediated NHEJ activities. The t–circle–tail structure resembles cyclized leading or lagging replication intermediates after excision by Topo–II from the genome. Inhibition of Topo–II cleavage activity by ICRF–187 decreases production of this extrachromosomal t–circle–tail. Similarly, inhibition of DNA–PK kinase or Lig4 activities decrease production of extrachromosomal t–circle–tail [76]. These results support a “looping–out” mechanism through ordination between topoisomerase II and NHEJ to resolve stalled replication fork at the telomeres (Fig. 2). This is consistent with our findings that DNA–PKcs is critically involved in the cellular response to replication stress, and it coordinates with the ATR signaling pathway for optimal replication checkpoint and fork recovery [75, 81, 111].

The circular types of extrachromosomal telomeric DNA, including double–stranded T–circles and the single–stranded C–circles, are commonly identified in cells with long telomeres through telomerase–mediated elongation. Cells with this characteristic include cancer and stem cell populations. These circular DNA types are associated with replication stress and the ALT mechanism [10, 112]. Excessive elongation of telomeres compromises telomere stability and is counterbalanced by a telomerase–trimming mechanism that involves a HR mechanism and production of extrachromosomal telomeric circles to maintain telomere homeostasis [113–115]. It is likely that the extensive telomeres are prone to replication stalling due to increased incidence of G–quadruplexes. The looping–out mechanism provide some explanations to the trimming of large telomeric segments and T–circle production in cells with long telomeres [76]. It is interesting to note that knockout of the Ku80 gene in human cells results in massive telomere loss due to HR–mediated t–circles and rapid deletion of the telomere, suggesting that the DNA–PK complex is essential for telomere homeostasis and cellular viability in human cells [62]. It is possible that DNA–PKcs–dependent NHEJ counterbalances and restricts the ability of the HR machinery to resolve stalled replication fork or DSB repair at telomeres. It is unclear whether the extrachromosomal telomeric circles are merely byproducts in resolving stalled replication forks or are actively involved in telomere maintenance. Telomeric circles have been suggested as the template for telomere extension by a rolling circle mechanism, under which the single–stranded C–circle serves as a template for the extension of the G–rich telomeric overhang [10].

DNA–PK could play additional roles in regulating the stability of telomeric G–quadruplex structure. For example, POT1 and hnRNP–A1 are capable of disrupting telomeric G–quadruplex [116, 117]. It is likely that DNA–PK kinase activity facilitates the removal of G–quadruplex through these telomeric DNA binding proteins during telomere replication. Alternatively, DNA–PK could influence G–quadruplex stability through RecQ helicases such as Wrn for telomere maintenance [118]. Notably, telomeric G–quadruplex also functions as a scaffold and is recognized by TLS/FUS (translocated in liposarcoma/fused in sarcoma) proto–oncoprotein through its C′ terminal RGG–rich domain, and that overexpression of TLS/FUS results in heterochromatin and telomere shortening in vivo [119]. It is possible that TLS/FUS binding stabilizes G–quadruplex structure and leads to progressive telomere shortening through hindering the completion of telomere replication. An independent study reported that TLS/FUS is a downstream phosphorylation target of DNA–PK [120], although it is not clear whether TLS/FUS phosphorylation by DNA–PK plays a role in telomere homeostasis regulation. Further investigation is needed to unveil DNA–PK’s impact on TLS/FUS regulation.

## Conclusions and future perspectives

The DNA–PK complex is crucial for telomere homeostasis regulation, particularly in human cells since depletion of the Ku heterodimer leads to severe telomere erosion and loss of cell viability. It is likely that the Ku heterodimer and catalytic DNA–PKcs subunit contribute to both overlapping and distinctive regulations to foster the integrity of telomeres, especially during telomere replication where they are involved in reestablishment of telomeric capping protection. The detailed mechanisms underlying DNA–PK promotion of telomere stability through protein–protein interactions and targeted phosphorylation remain to be elucidated.

Loss of DDR regulators is commonly associated with genomic instability and cancer development [121, 122]. On the contrary, overexpression of DNA–PKcs seemingly occurs in many cancer types [123]. Whether DNA–PKcs overexpression contributes to telomere homeostasis during carcinogenesis requires further study. Nonetheless, a combination of anti–DNA–PKcs and anti–telomere strategies have proposed and might offer additional tools in combatting aggressive and radioresistant tumors [124–127]. Further investigation will help to determine the benefit of these combined modality approaches to cancer patients.

## Data Availability

Not applicable.

## Abbreviations

ATM: Ataxia–telangiectasia mutated
ATR: Ataxia–telangiectasia and Rad3–related
Chk1: Checkpoint kinase 1
DNA–PK: DNA–dependent protein kinase
DNA–PKcs: DNA–dependent protein kinase catalytic subunit
DSBs: DNA double–strand breaks
HR: Homologous recombination
Ku: Ku70/80
NHEJ: Non–homologous end–joining
PI3K: Phosphatidylinositol–3 kinase
PIKK: Phosphatidylinositol–3 kinase–like kinase
POT1: Protection of telomeres 1
RAP1: Repressor activator protein 1
Terc: Telomerase RNA component
Tert: Telomerase reverse transcriptase
TIN2: TRF1–interacting nuclear protein 2
TLS/FUS: Translocated in liposarcoma/fused in sarcoma
TPP1: Telomere protection protein 1
TRF1: Telomeric repeat–binding factor 1
TRF2: Telomeric repeat0binding factor 2
